# HAPPI-2: a Comprehensive and High-quality Map of Human Annotated and Predicted Protein Interactions

**DOI:** 10.1186/s12864-017-3512-1

**Published:** 2017-02-17

**Authors:** Jake Y. Chen, Ragini Pandey, Thanh M. Nguyen

**Affiliations:** 1 Wenzhou Medical University First Affiliate Hospital, Wenzhou, Zhejiang Province China; 2 Medeolinx, LLC, Indianapolis, IN 46280 USA; 3 The Informatics Institute, University of Alabama at Birmingham School of Medicine, Birmingham, AL 35294 USA; 4 Indiana Center for Systems Biology and Personalized Medicine, Indiana University School of Informatics and Computing, Indianapolis, IN 46202 USA

## Abstract

**Background:**

Human protein-protein interaction (PPI) data is essential to network and systems biology studies. PPI data can help biochemists hypothesize how proteins form complexes by binding to each other, how extracellular signals propagate through post-translational modification of de-activated signaling molecules, and how chemical reactions are coupled by enzymes involved in a complex biological process. Our capability to develop good public database resources for human PPI data has a direct impact on the quality of future research on genome biology and medicine.

**Results:**

The database of Human Annotated and Predicted Protein Interactions (HAPPI) version 2.0 is a major update to the original HAPPI 1.0 database. It contains 2,922,202 unique protein-protein interactions (PPI) linked by 23,060 human proteins, making it the most comprehensive database covering human PPI data today. These PPIs contain both physical/direct interactions and high-quality functional/indirect interactions. Compared with the HAPPI 1.0 database release, HAPPI database version 2.0 (HAPPI-2) represents a 485% of human PPI data coverage increase and a 73% protein coverage increase. The revamped HAPPI web portal provides users with a friendly search, curation, and data retrieval interface, allowing them to retrieve human PPIs and available annotation information on the interaction type, interaction quality, interacting partner drug targeting data, and disease information. The updated HAPPI-2 can be freely accessed by Academic users at http://discovery.informatics.uab.edu/HAPPI.

**Conclusions:**

While the underlying data for HAPPI-2 are integrated from a diverse data sources, the new HAPPI-2 release represents a good balance between data coverage and data quality of human PPIs, making it ideally suited for network biology.

**Electronic supplementary material:**

The online version of this article (doi:10.1186/s12864-017-3512-1) contains supplementary material, which is available to authorized users.

## Background

Human protein-protein interactions (PPI) has become a fundamental data type to biomedical systems biology research areas such as “network biology” and “network medicine” [1, 2]. PPI data can help biochemists hypothesize how protein complexes form by binding to each other [3, 4], how extracellular signals propagate through post-translational modification of signaling molecules [5, 6], and how chemical reactions are coupled together in a complex biological process [1]. PPI data can also help genome scientists build gene network modules in the analysis of large amount of next-generation sequencing data to identify functionally significant genomic variations among tens of thousands of candidate measured signal changes [7, 8]. PPI data can also help systems biologists develop better disease diagnostic and prognostic biomarkers by linking candidate biomarkers into “stable modules” [2, 9] than by using single gene or protein as “biomarkers”, a common practice that often suffers from lack of specificity and robustness. Moreover, PPI data can help drug developers prioritize drug target selections based on ​newly characterized network topological properties, e.g., PPI network centrality measures of genes, in a disease gene network  [10–14], or by designing drugs to “pick the pocket” of proteins targeting critical PPI interfaces as a new drug development strategy [15]. Our capability to develop comprehensive high-quality public human PPI databases has a direct impact on future research on genome biology and medicine [16].

The surging interest to incorporate PPI data into a wide range of biomedical studies is complicated by the fact that there is still incomplete coverage of available human PPI data reported today. Since the first report of the initial large-scale human protein interaction of 13,656 in 2003 by Chen *et al* [17] and a draft public data release of 70,000 physical interactions in 2004 [18], the number of reported human PPIs has grown steadily. In 2009, we reported in the HAPPI database release 1.0 (HAPPI-1) a catalogue of more than 140,000 medium-to-high-confidence human PPIs [19]. In mid-2014, this data set was surpassed by the BioGRID database to reach approximately 268,599 curated physical and genetic interactions [20]. In spite of the data growth, Stumpf et al. estimated the entire human protein interactome size to be approximately 650,000 [21], assuming these PPIs are primarily based on physical binding. The STRING database tried to overcome the limited PPI data coverage issue through comprehensive collection of known and predicted protein interactions, which include 2,132,575 direct (physical) and indirect (functional) associations for human. Apparently, the contradiction in counts highlight the challenge in both predicting human PPI data and separating physical and functional PPI data. While research on predicting human PPI types from transient to stable binding is still ongoing [22], there has been concerns on how to balance PPI data coverage and quality. In practical applications, bioinformatic scientists tend to favor the inclusion of more functional interaction data to boost network data coverage while biologists tend to trust only strong physical interactions for signaling network constructions [23]. In addition, researchers favor PPI databases with categorical classifications that express how closely the two proteins are related functionally or physically during an PPI event than those without such information [24–26]. This demand drives ongoing efforts in human PPI data integration and annotation.

In this work, we describe HAPPI (Human Annotated and Predicted Protein Interactions) database release 2.0 (HAPPI-2), accessible to the public at http://discovery.informatics.uab.edu/HAPPI. HAPPI-2 is a major update to the original HAPPI-1 database, which has been indexed since 2009 by the PathGuide, a comprehensive online pathway data resource guidebook [27]. HAPPI-1 generated a wide range of biomedical research applications, including: drugs’ side-effects discovery [28], protein isoform identification [29], pathway development [30], biomarker discovery for diabetes [7], Hepatocellular Carcinoma biomarker expression analysis [31], etc. In this release, we compiled human PPIs from a wide variety of experimental and computational methods, which include both direct physical interactions and functional associations derived from multiple platforms such as microarrays, affinity purification, yeast two-hybrid, co-expression, similar sequences, genome context, and homology-based PPI inference [32–39]. Compared with HAPPI-1, the human PPI data coverage (at all confidence levels) in HAPPI-2 has increased by almost five-fold from 604,741 (for HAPPI-1) to 2,922,202 (for HAPPI-2) entries, among which 640,798 are of medium-to-high-confidence PPIs. The coverage of unique and curated UniProt protein entries in HAPPI has also expanded from 13,601 for HAPPI-1 to 23,464 for HAPPI-2. In HAPPI-2, human PPIs are categorized similarly to the HAPPI-1 database into five confidence quality ratings, i.e., from 1-star (based on predicted and likely functional associations) to 5-star (enriched by curated and likely physical associations), as determined jointly by PPI data’s different sources, data generation methods, and available literature references. These confidence quality ratings of PPIs are validated with two complementary methods, one by assessing the statistic of shared gene ontology similarity score among curated PPI pairs and the other by assessing the percentage of conserved MetaGene interaction pairs. We also redesigned HAPPI-2 web portal to make it easy for biology researchers to query, browse, annotate, store, batch retrieve, and curate medium-confidence to high-confidence human PPIs. The growing list of advanced features include: searching the database with multiple human gene/protein identifiers, annotating proteins involved in PPIs with drug targets and disease relevance information, and limited user annotation functions for specific PPI of interest. The updated HAPPI-2 web portal provides a uniform, quality-rated, searchable, and annotated online resource of human PPI data for biomedical researchers interested in network biology applications.

## Method

### Source and coverage of human PPI data

Human PPI data in the HAPPI-2 database are compiled from the following database sources (with final counts reported after mapping all gene symbols and protein IDs to corresponding reviewed UniProtKB ID): HAPPI v1.1, which consists of both annotated and predicted human interactions; BioGRID v3.2 [40], which consists of 219,178 physical and genetic interactions; IntNetDB v1.0 [41], which consists of 306,442 PPIs integrated using a probabilistic model; I2D v2.3 [42], which consists of 236,541 PPIs integrated or predicted for human; STRING v9.1 [43], which consists of 2,166,793 both known physical and predicted functional interactions for human; HPRD v9 [44], which contains 27,282 manually curated interactions; and Wang’s human molecular signaling data set v6 [45], which consists of 48,945 manually curated human molecular signaling data. After downloading the source data, raw data files were parsed using Python scripts first and processed data were uploaded into an Oracle 11G relational database using the provided SQLLDR utility. All the human proteins are mapped to their UniProt identifiers using reviewed subset of the UniProtKB for standardized representations in the new HAPPI database.

### PPI data confidence scoring and categorization

We extended the unified PPI data scoring scheme, which we initially described in HAPPI-1 [19], to encode the likelihood that a human PPI of interest may arise from physical and direct relationships of each other instead of from functional and indirect relationships. The extended scoring framework can be described as the following. First, we rated each PPI experimental/computational platform using a reliability index (a value between 0 and 1) similar to that in HAPPI-1. The more trustworthy we have for an experimental or computational platform to detect direct/physical interactions, the higher (up to 1) the reliability index value we assign to the platform. Second, we integrate individual reliability index values for each PPI over different platforms into a final score *P*_*II*_, using the following formula:

1 $$ {P}_{II}=1-{\displaystyle {\prod}_{i=1}^N\left(1-{S}_i\right)} $$

where *S*_*i*_ is an independent score for the PPI of interest based on platform *i* and *N* is the number of different platforms curated for the PPI. *P*_*II*_ should range from 0 to 1. Third, we further integrate available PPI confidence score from HAPPI-1 (*P*_*I*_) with the new PPI confidence score calculated for HAPPI-2 (*P*_*II*_) using the following calculation to derive the final score ***P***:

2 $$ \boldsymbol{P}=1-\left(1-{P}_I\right)\ast \left(1-{P}_{I I}\right) $$

The primary reason for combing the confidence scores this way is to ensure that a) current update does minimal impact to the scoring scheme and b) future upgrade may be performed only locally for those when new information becomes available. Therefore, this scheme can be applied to all future scoring updates from the current release (by using P_I_) and the future release (by using P_II_). Here, PPIs unique to HAPPI-2 are set to a default value of *P*_*I*_ =0. To categorize the confidence score into 5-star confidence levels in HAPPI-2, we used the same following *P* threshold ranges that were originally defined in HAPPI-1:

- 1-Star (Ultra-low confidence): *P* < 0.25
- 2-Star (Low confidence): 0.25 ≤ *P* < 0.45
- 3-Star (Medium confidence): 0.45 ≤ *P* < 0.75
- 4-Star (High confidence): 0.75 ≤ *P* < 0.90
- 5-Star (Ultra-high confidence): 0.90 ≤ *P* ≤ 1

### Data annotation and user curation

In HAPPI-2, we curated the human PPI data with the following information: known sources of the PPI, known association/binding types, known effect of association such as inhibition or activation, and PPI data confidence category ratings. All proteins involved in these PPIs were also annotated with key functional information such as protein function, pathway involved, protein family, disease implication, and targeting drugs. The annotation data were collected from UniProt [46], GenBank [47], Pfam [48], and DrugBank [49], Gene Ontology (GO) [50], PDB [51], and the HPD/PAGED pathway databases [30, 52] and subsequently imported into the HAPPI-2 database backend. On the web interface, we also provided users with URLs that link out from HAPPI-2 PPI records to the source web sites. Of particular mention is the user-specific PPI curation experimental feature, in which we provide users with the new ability to rank and comment specific PPIs from their logged accounts. Users can either “like” or “dislike” any retrieved PPI to keep track of PPI deemed as “valid” or “invalid” by themselves. They can also add additional PPI interaction details to share to the public.

### PPI data quality assessment

While it can be challenging to establish golden standards for both true positive and true negative human PPIs due to the plurality of PPI types, current research findings generally focus on two aspects of data quality assessment: 1) stochastic errors, which may arise from false positives or false negatives during the data collection process, and 2) systematic errors, which may arise from potential biases in human PPI experimental data generation or integrated data collection. Stochastic errors may be evaluated using biological validation frameworks such as 3D complex structure confirmation [53], shared GO term count [53], and co-expression correlations [54, 55], by comparing the PPI data set with a randomly generated PPI data set. Systematic errors may be assessed using statistical evaluations of the proteins represented in the PPI data set [56, 57].

1. Evaluation of stochastic errors
  We adopted two approaches to evaluating HAPPI-2 PPI data’s stochastic errors. The first approach uses a MetaGene pair enrichment (MPE) technique that we developed earlier in HAPPI-1. The method uses evolutionarily conserved co-expression pairs to assess protein interaction quality, which we defined as the probability to return validated PPIs from retrieved results. Note that we cannot use established True Positive Rate or False Positive Rate concepts, because it is still fairly uncharacterized in this field what human PPIs are true positives and true negatives, even in the literature curated database [58]. While many PPI data sets were cross-validated with species-specific gene co-expression profiles [55], co-expression correlation alone has proven to be less reliable in characterizing PPI data quality or PPI network properties [59]. Therefore, methods using evolutionary-conserved species-neutral co-expression of orthologs of interacting partners such as [55] have been proposed. Such methods are shown to be more sensitive overall to predict or validate PPIs than those using information purely from the organism, e.g., simple co-expression, cellular co-localization, or functional category enrichment similarity [54]. For this purpose, we used MetaGene [60], a comprehensive evolutionarily conserved co-expressed gene data set by Stuart et al, to independently evaluate the interactions from different databases using the PPI data quality metric defined in similar evaluation studies in HAPPI-1. The MetaGene data set involves 6,591 human genes and 22,154 evolutionary conserved co-expression relationships from humans, flies, worms, and yeast, based on the analysis of over 3182 published DNA microarray experiments. In this work, we decided to use the MetaGene database instead of the other newer and more comprehensive databases, such as CoXPRESS [61], the Human Gene Coexpression Database [62], due to the conservation of co-expression across multiple species. In essence, we take a random sample of a PPI database of interest, with each sample consisting of 1000 PPIs, to characterize the sample’s overlap with the entire MetaGene PPI data set. Then, we repeat the random sampling and above sample overlap analysis for 1000 times to obtain a distribution of sample counts over binned overlapping count ranges. The distributions for different PPI databases, varying by different filter conditions, will be compared with each other for the distribution’s means and spread statistic. The higher the value of the distribution mean and the better separated the distribution from the other in comparison, the better quality of the PPI data sample has. Since we do not incorporate MetaGene in our database or its constituent database during HAPPI-2 development, the concern for introducing evaluation bias with this method is minimal. Note that when comparing databases of different sizes, we also introduce the concept of “normalized sample size” for determining the randomized sampling size against the size for HAPPI-1 data. Normalization is necessary for a fair comparisons of the overlap results between HAPPI data sets of varied sizes, because the MetaGene database is fixed in size and we use all its contents to overlap with the database subset during sampling comparisons.
  The second approach uses Gene Ontology (GO) term similarity (GOS) index, which is widely used to test PPIs with well-characterized functions but not those with novel functions [63]. We included this approach primarily to provide supplemental perspective of the first approach. In the GOS approach, we first form a sample consisting of randomly selected 1000 PPIs for each confidence level (from 1-star to 5-star categories). Then, we determined the statistical distribution of the GO term similarity index among all PPIs in each sample, which is calculated by using the funSim algorithm [64] from the GOSim package v.3.0 [65]. Lastly, we performed a *t*-test to evaluate the differences between each PPI sample and a randomly generated negative PPI data set. A *p*-value is provided for each pair of comparisons.
2. Evaluation of systematic errors
  We evaluate systematic errors of the PPIs in our database using established gene/protein functional enrichment analysis in bioinformatics. To establish gene/protein functional enrichment, we used the DAVID [66] bioinformatics toolset to obtain interacting protein’s functional annotation charts and functional annotation clustering tool, using DAVID’s default parameters before reporting the names of enriched GO [50] functional categories in molecular functions and biological processes subsets, using Benjamini’s adjusted *P*-value threshold of 0.05. To compare between HAPPI-1 and HAPPI-2, we plot histograms using the count of functional annotation categories identified or the count of functional annotated cluster separately.
  To focus on evaluating systematic errors of the data set, we use three performance measures. First, we evaluate the *Absent Protein Bias*, which may be observed by the enrichment analysis of proteins that are reported in the UniProt database (curated portion) but absent from the human PPI database of interest. Second, we evaluate *Missing Overlap Bias*, which may be observed by the enrichment analysis of proteins in MetaGene that are from the non-overlapped portion of the MetaGene, when we perform a global PPI quality assessment by overlapping the whole human PPI database of interest with MetaGene pairs. Third, we evaluate *Hub Enrichment Bias*, which may be observed by the enrichment analysis of proteins that appear in the top-100 well connected proteins by their degree of connectivity. Overall, when observation of any of the three types of biases are noted—while still incomplete to address the human PPI data quality issue in its entirety—we will nonetheless gain an improved understanding how the integrated human PPI database of interests stands against comparable databases for data coverage biases.
3. Evaluation of false positive errors
  We evaluated false positive errors of the PPIs in our database by the overlapping between our HAPPI-2 database and the manual-stringent section of the Negatome 2.0 database [67]. Negatome’s mannual stringent section contains 1991 manually curated pairs of protein which do not physically interact excluding interactions detected by high-throughput approaches. Similar to other PPI databases included in HAPPI-2, we mapped protein identification in Negatome to UniProt identification to acquire the overlapping interactions between HAPPI-2 and Negatome. We defined the false positive ratio of HAPPI-2 by
  3 $$ E= O/ N $$
  Where ***O*** is the count of overlapping PPIs between HAPPI-2 and Negatome 2.0 and ***N*** is the size of HAPPI-2 database. To show the improvement of HAPPI-2, compared to HAPPI-1 and STRING, in false positive errors, we applied formula (3) on HAPPI-1 and STRING with different reliability index (for HAPPI) and scale-to-1 confidence score [68] (for STRING). In addition, considering Metagene’s interactions as the true positive set and Negatome’s manual-stringent interaction as the true negative set, we calculated the area-under-curve (AUC) using the reliability for HAPPI-2/HAPPI-1 and confidence score for STRING.

### Comparative evaluation of PPI data coverage, quality, and network property

We applied several parameters to evaluate the human PPI data coverage and data quality between the new HAPPI-2 database and a few other underlying human PPI databases in comparison. We define a database coverage overlap ratio, r_*i,j*_, as the following:

4 $$ {r}_{i, j}={O}_{i, j}/{N}_i $$

Where O_*i,j*_ represents the human PPI data overlap count between database *i* and database *j*, and N_*i*_ is the size of the database *i*. ${r}_{i, j}$ reported will indicate how much redundant information exists between two database sources in the integrated HAPPI-2 database. We define a database global data quality score, *q-score*, as the overlap between the MetaGene pairs and the database’s all human PPIs. For the database’s PPI network properties, we refer to PPI network properties such as the node degree of connectivity, node centrality, clustering coefficient, network diameter (for standard definitions, refer to [69]), all within the largest connected network that can be constructed from the human PPI database of interest. Here, we used Stanford Network Analysis Platform (SNAP) library [70] to analyze connected networks for each database of human PPIs using network characteristics. To evaluate the ranking of top-100 hub proteins for the database of interest, in which “hub” is defined by the node degree of connectivity and rank is given by descending orders of node degree of connectivity for all proteins in the database, we used Spearman’s rank correlation coefficient ρ, which can be computed from the following:

5 $$ \rho =1-6{\displaystyle {\sum}_i{d}_i^2/ n\left({n}^2-1\right)} $$

Where d_*i*_ is the difference between two ranks of top-100 hub proteins, each of which comes from a different database source under comparison.

### Designing case study: database validation through PPI rediscovery

**Fig. 1:**
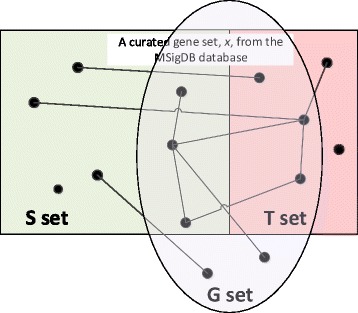
An illustration of how we define the “database validation through PPI rediscovery” approach. The rectangle area consisting of both the S set (in *green shades*) and the T set (in *red shades*) represents all genes taken from a curated gene set *x* from the MSigDB database. The lines connecting genes are PPIs all taken from a single database under validation. The G set shows new (“discovered”, outside of the rectangle) or old genes (“re-discovered”, in either the S set or the T set) by expanding from genes in the S set by one layer of PPI relationships

We designed the Missing Genes Retrieval in Curated Gene Sets using PPIs problem as a case-study to compare the biological significance of HAPPI-2 database. We randomly partitioned each curated gene set into the seeded set (S) and hidden set (H). From the seeded set, we queried the PPI databases to acquire the one-step-expansion gene set (G), which contained every gene directly interacting to genes in S. With the G set, we computed the retrieval sensitivity by |H∩G|/|H|, where |H| is the number of genes in H and |H∩G is the number of genes overlapping between H and G (see Fig. 1 for more detail). To further explore the impact of PPI database expansion, which tends to inflate the G set, in improving rediscovery, we design the rediscovery factor *α* as follow:

6 $$ \alpha =\left|\mathrm{H}\cap \mathrm{G}\right|/\left|\mathrm{G}\right|\times f $$

where *f* is the adjusted expansion factor depending on PPI database. For BioGRID database, f is always 1. For other non-BioGRID databases we set f as the ratio between the size of G-set acquired by the database and the size of G-set acquired by BioGRID. In this problem, we used 186 curated KEGG gene sets with size of at least 10 from MSigDB database [71]. We used gene set size filtering to ease the random partition process. For each gene set, we repeat the experiment 50 times to ensure the statistical significance of the result.

### Web-based database application development

We designed the HAPPI-2 database using a relational database schema and implemented it using the Oracle DBMS. The database application adopts a three-tier application architecture. The database tier is hosted on the Indiana University’s high-performance computing facility and professionally managed. The middle tier runs the Apache web server and the middleware software serving data from the database to web clients were written in Perl and php. The front-end tier runs on any modern web browser, with Javascript and CSS technologies to enhance user interactions. The infrastructure can scale up to accommodate medium Internet traffic. To access the HAPPI-2 web application and details on the database and software development documentation, user guides, and frequently asked questions, users can visit http://discovery.informatics.uab.edu/HAPPI.

## Results

### Content coverage

We have developed the HAPPI-2 database to include 2,922,202 distinct human PPIs, the largest integrated compilation of human PPIs today. This represents a 485% of the original count of PPIs in HAPPI-1 and also a significant increase of coverage over BioGRID or STRING. HAPPI-2 covers PPI information among 23,060 human proteins identified by their UniProt IDs—an increase of approximately 12% over HAPPI-1. The count of medium-confidence and above (3-, 4-, and 5-star) PPIs have increased from 142,523 for HAPPI-1 to 640,748 for HAPPI-2 (Table 1). The extent of overlap among HAPPI-2’s constituent source databases are shown in Table 2, which show why a comprehensive database integration effort such as HAPPI-2 is needed. The overlap analysis shows that, despite various ongoing PPI data integration projects, there are still significant room for PPI data coverage improvement.

**Table 1:** A comparison of human PPI data coverage distributed over several sub-categories

	HAPPI-1	HAPPI-2	Increase ratio
All Interactions	604,741	2,922,202	483%
5-star	37,754	175,476	464%
4-star	33,733	167,123	495%
3-star	71,036	298,149	419%
2-star	189,150	854,189	452%
1-star	273,068	1,427,265	523%

**Table 2:** Human PPI data coverage overlap (in %) among its constituent database sources

	HPRD	I2d	IntNetDB	Wang	BioGRID	STRING
HPRD	100.00	67.21	1.13	14.38	41.34	58.56
I2d	15.50	100.00	1.49	5.78	26.00	38.42
IntNetDB	0.10	1.15	100.00	0.45	0.97	16.19
Wang	16.03	27.95	2.80	100.00	24.87	92.33
BioGRID	10.29	28.06	1.36	5.55	100.00	43.01
STRING	0.15	0.42	0.23	0.21	0.44	100.00

The percentage number are the overlapping count between the two data sources at the intersection of row and column divided by the size of data source for the row

### Data functional and network characteristics

**Fig. 2:**
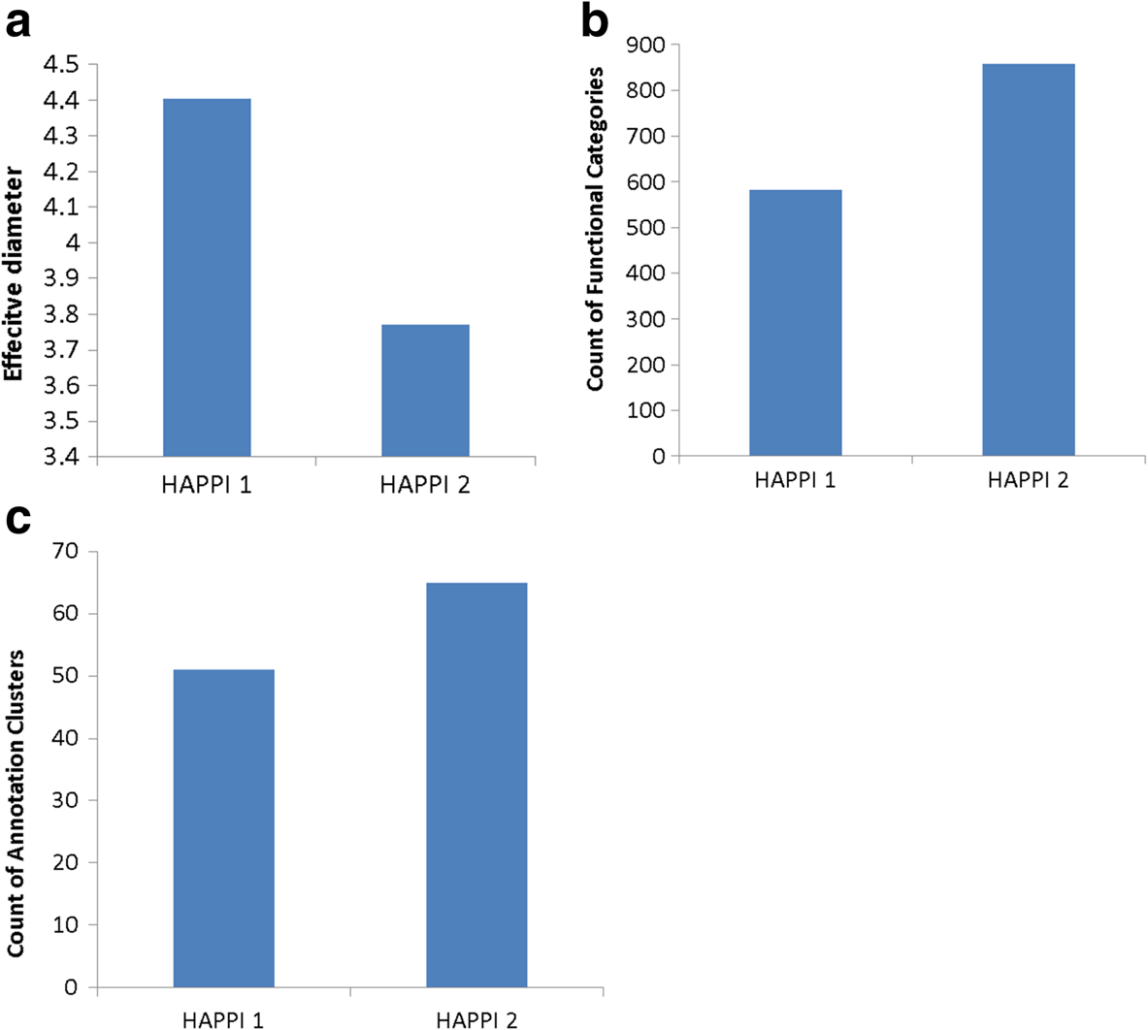
A comparison between HAPPI-1 and HAPPI-2 for human PPIs with a confidence rating of 4-star and above. **a** Effective diameter. **b** Covered Functional Categories. **c** Covered Annotation Clusters

In addition to the increase in PPI data coverage, the PPI network density and PPI functional category diversity—features often desirable for advanced network biology studies [7, 72, 73]—have also improved with the new release of HAPPI-2. In Fig. 2a-c, we show that there is a significant reduction of HAPPI-2 network effective diameter [69] and significant expansion of functional annotations and functional automation clusters observed with the DAVID analysis of high-confidence (4- and 5-star confidence ratings) interacting proteins obtained. Here, a *network diameter* represents an important metric to evaluate closeness of nodes in a large complex network (see the Method section); the smaller the value is, the more highly connected the PPI network becomes. The results from Fig. 2 also confirmed that the expanded interaction protein coverage for high-quality PPI subsets led to increased protein functional category diversity in HAPPI-2 over HAPPI-1: 47% increase in number of functional categories and 29% increase in number of annotated clusters.

**Fig. 3:**
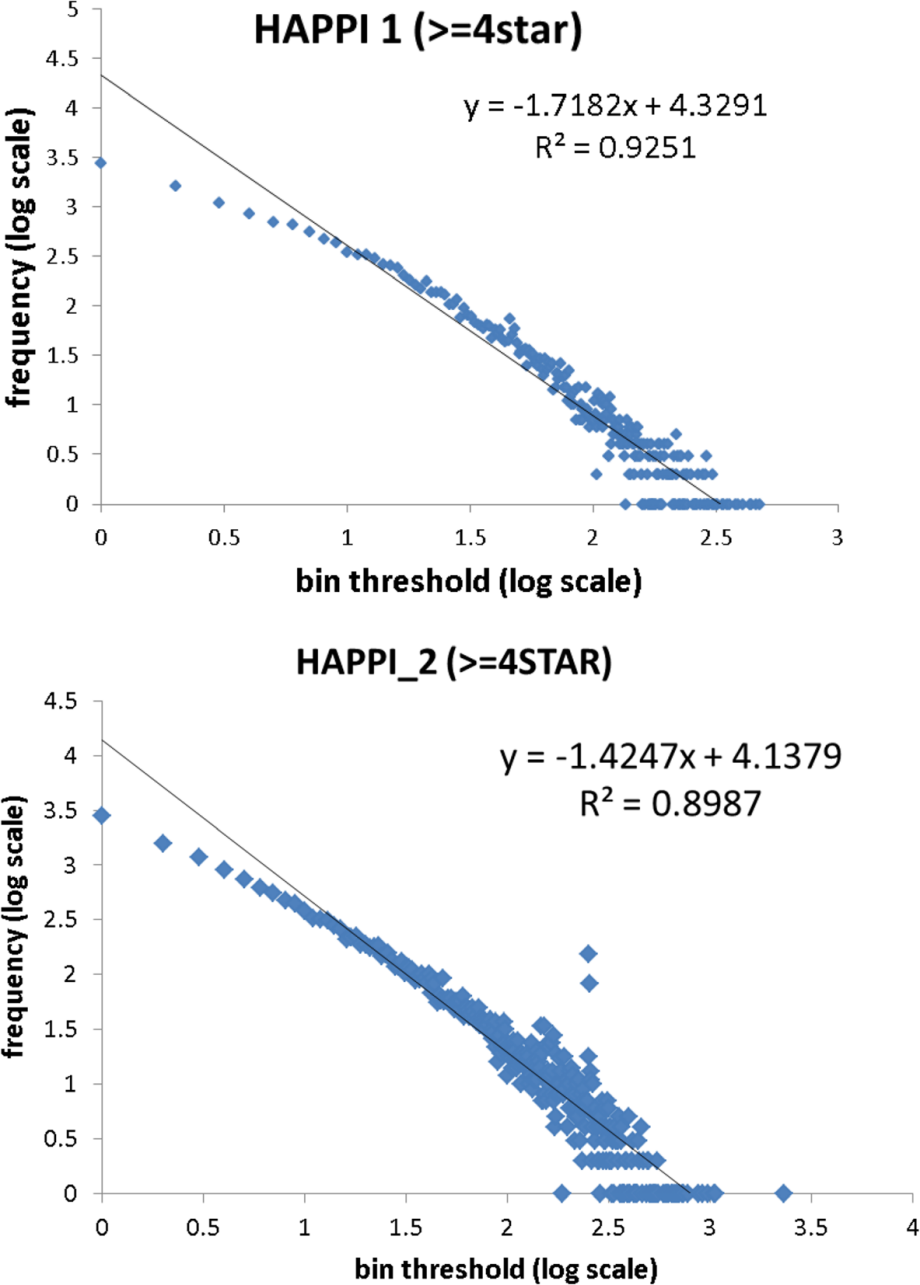
A comparison of protein network degree distribution between HAPPI-1 and HAPPI-2

In Fig. 3, we show a comparison of the human PPI data’s scale-free characteristics between HAPPI-1 and HAPPI-2 among high-quality PPIs, i.e., with 4-star and 5-star confidence ratings. The two sets of data showed similar intercept and regression R^2^ for the linear function in the node degree distributions plotted using log-log scales, with HAPPI-2 having slightly flatter slopes. This not only confirms the scale-free property for both data sets, but also confirms the trend as suggested by reduced network diameter (Fig. 2a) for the updated HAPPI-2 to have “network hubs” with higher degree of connectivity than HAPPI-1 as the data coverage is expanded.

### Data quality evaluations

**Fig. 4:**
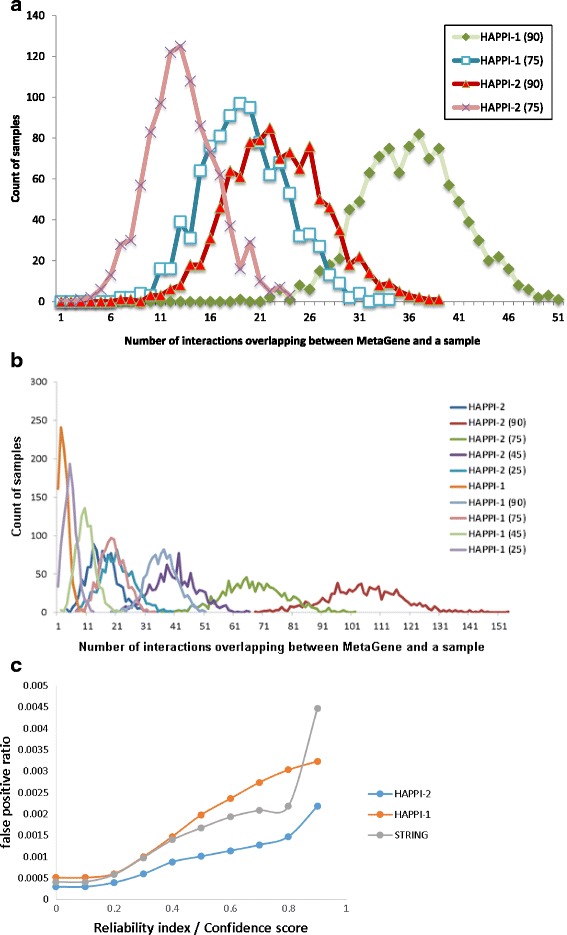
**a** A comparison of distributions of MetaGene overlap counts for randomized samples (size = 10,000 per sample) of HAPPI-1 and HAPPI-2 database subsets. **b** A comparison of distributions of MetaGene overlap counts for randomized samples of HAPPI-1 and HAPPI-2 database subsets. The results shown has all randomized samples normalized with the database size differences between HAPPI-1 and HAPPI-2 databases, currently with a size ratio factor of 1:4.85. **c** A comparison of false positive ratio among HAPPI-2, HAPPI-1 and STRING. Here, the STRING’s confidence score (scale 1000) is scaled to 1 to compare with HAPPI-2’s and HAPPI-1’s confidence score on the x-axis (or reliability index for data from a single source)

In Fig. 4a, we showed an assessment of each HAPPI database subsets for their tendency to contain true positive PPI data, by overlapping the MetaGene golden standard data with randomized samples of un-normalized size = 10,000 each (see Methods for details) for HAPPI-1, HAPPI-2 ultra-high-confidence (confidence score ≥ 90%) and high-confidence (confidence score ≥ 75%) subsets. The two-digit numbers shown in parenthesis in the data series legend for the database HAPPI-1 or HAPPI-2 refer to the minimal PPI confidence quality scores (in percentage scale) used to construct the data subset. For example, “HAPPI-2(90)” refers to the HAPPI-2 database subset with ultra-high confidence, in which all the PPI data has a confidence score ≥ 90%. Keep in mind that the size of HAPPI-2 is approximately 4.85-fold of that for HAPPI-1 (Table 1) and that the MetaGene golden standard data set is of fixed size and used entirely for the overlapping count analysis. The distributions show approximately the same range and trend that we observed and validated for the HAPPI-1 in its initial publication. The small shift to the left side for the HAPPI-2 (90) and HAPPI-2 MetaGene overlap count distributions compared to the corresponding ones for the HAPPI-1 (90) and HAPPI-2 can be explained by the 4.85-fold size differences between HAPPI-2 and HAPPI-1. When we choose a random sample size adjusted to the 4.85-fold change between HAPPI-2 and HAPPI-1 database, the HAPPI-2 shows a significantly higher tendency than HAPPI-1 to become validated with the MetaGene pairs across all confidence quality categories (Fig. 4b). In other words, we demonstrated that there are more validated MetaGene pairs in HAPPI-2 than those in HAPPI-1 for the same slice of the respective database.

In Fig. 4c, we showed that HAPPI-2 acquired less false positive ratio (described in formula (3)) than STRING and HAPPI-1. However, when examining the counts of overlapping interactions between HAPPI-2/HAPPI-1/STRING and Negatome’s manual-stringent subset (NMS), we found that these PPI databases above significantly overlap with the Negatome’s manual-stringent subset. Over 1,991 interactions in NMS, HAPPI-2 shared 871 interactions, HAPPI-1 shared 309 interaction and STRING shared 894 interactions. Surprisingly, when we applied the same process for the BioGRID database, we found that BioGRID and NMS shared 401 interactions, which counts for 20.14% of the NMS. These facts explain why AUCs using both HAPPI’s reliability index and STRING’s confidence score to classify true positive PPIs (with MetaGene) versus true negative PPIs (with NMS) are low. HAPPI-2 only acquired AUC of 0.477 and STRING only acquired AUC of 0.483.

**Fig. 5:**
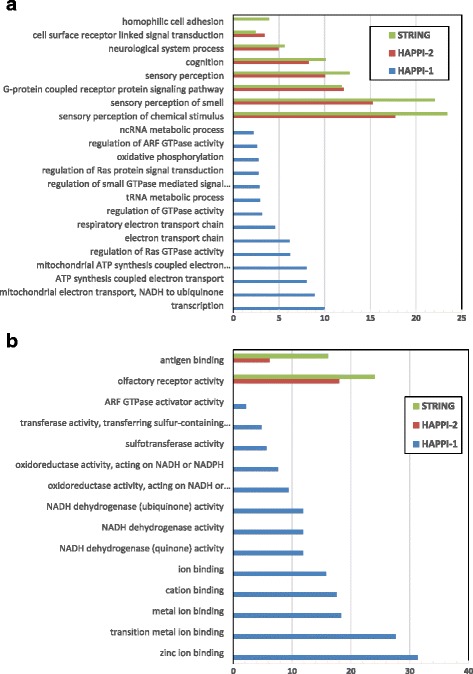
**a** A comparison of protein functional category enrichment as of a result of “Absent Protein Bias” among three human PPI databases. The x-axis shows the –log(*p*-value) returned by DAVID for enriched functional categories of proteins under examination for absent protein biases. Results are shown for A) GO Biological Processes subcategories. **b** A comparison of protein functional category enrichment as of a result of “Absent Protein Bias” among three human PPI databases. The x-axis shows the –log(*p*-value) returned by DAVID for enriched functional categories of proteins under examination for absent protein biases. Results are shown for B) GO Molecular Function subcategories

**Fig. 6:**
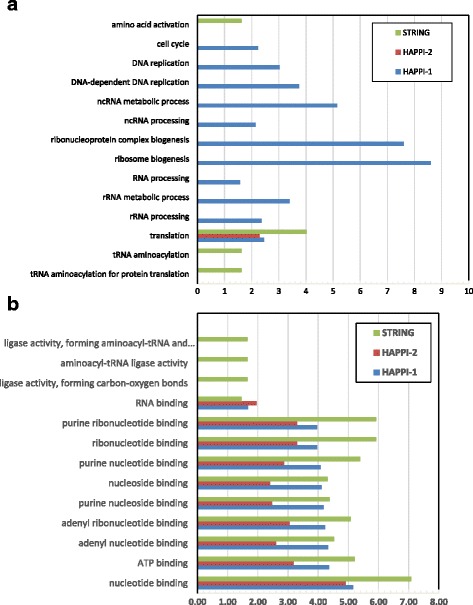
**a** A comparison of protein functional category enrichment as of a result of “Missing Overlap Bias” among three human PPI databases. The x-axis shows the –log(*p*-value) returned by DAVID for enriched functional categories of proteins under examination for missing overlap biases. Results are shown for A) GO Biological Processes subcategories. **b** A comparison of protein functional category enrichment as of a result of “Missing Overlap Bias” among three human PPI databases. The x-axis shows the –log(*p*-value) returned by DAVID for enriched functional categories of proteins under examination for missing overlap biases. Results are shown for B) GO Molecular Function subcategories

To evaluate systematic biases, we particularly examined both the “absent protein bias” and “missing overlap bias” among three human PPI databases, STRING (v9.1), HAPPI-1, and HAPPI-2. STRING was chosen for its widespread popularity and high data coverage close to that of HAPPI-2. We show our analysis of these examination results in Figs. 5 and 6 next.

In Figs. 5a and b, we show comparisons of protein functional category enrichment as a result of “absent protein bias” in GO Biological Processes category and GO Molecular Function category respectively. The categorical distribution and length of protein enrichment histogram bars for each of the three databases suggest the extent of the presence of the “absent protein bias”. These results show that HAPPI-2 overall has the least amount of “absent protein bias” in both molecular functions and biological processes among the three databases compared. For the biological processes subcategory, both HAPPI-2 and STRING seem to lack sufficient coverage of proteins involved in important biological processes such as sensory and extracellular cell signaling. These problems highlighted the limitations of current human PPI data collection efforts. HAPPI-1, on the other hand, has a quite different profile for the “absent protein bias” that is generally broader than those found for HAPPI-2 and STRING. For the molecular function subcategory, the “absent protein bias” issue seem to be significantly less severe for HAPPI-2 and STRING than for HAPPI-1. Again, we observed lack of protein coverage in human PPIs for antigen binding and olfactory receptor activity function.

In Fig. 6a and b, we show comparisons of protein functional category enrichment as a result of “missing overlap bias” in GO Biological Processes category and GO Molecular Function category respectively. The categorical distribution and length of protein enrichment histogram bars for each of the three databases suggest the extent of the presence of the “missing overlap bias”, the parameter that gauges potential false negative human PPIs in the human PPI database. These results show that HAPPI-2 overall has the least amount of “absent protein bias” in both molecular functions and biological processes among the three databases compared. For the biological processes subcategory, HAPPI-1 seem to have missed many proteins involved in cell cycle, RNA replication, and various types of RNA processing. This was addressed properly in the HAPPI-2 update. All three databases still have the “missing overlap bias” of varying degrees in the “translation” category nonetheless. For the molecular function subcategory, the “missing overlap bias” issue seems to be more prevalent and consistent with one another, with the majority of the biases concentrated on non-protein binding categories. For PPI data, this observation can be attributed to the protein-binding data coverage bias inherent in the biology.

**Fig. 7:**
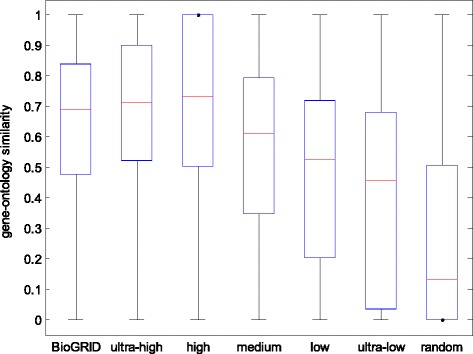
GO similarity from ultra-high-confidence PPIs to ultra-low-confidence PPIs, in comparison with BioGRID PPIs and random PPIs

In Fig. 7, we show the significant difference on GO term similarity among interactions from different categories. First, in the entire HAPPI-2, the medium to ultra-high-confidence interactions (three star and above) achieves higher GO term similarity than low-ultra-low-confidence interactions (*p*-value = 3.29 × 10^−78^). Second, the GO similarity scores of both the medium to ultra-high confidence PPIs and the ultra-low-confidence PPIs are significantly superior to the GO similarity scores of random PPIs, which support our claims on HAPPI-2’s quality. Third, the GO similarity scores of HAPPI’s medium-confidence PPIs are similar to the ones of BioGRID’s PPIs.

### Performance of HAPPI databases in database validation through PPI rediscovery

Comparing the performance among HAPPI-2, STRING and BioGRID in the validation through PPI rediscovery problem, we claim the biological significance of HAPPI-2. The HAPPI-2’s sensitivity (mean = 0.809) is higher than the STRING’s sensitivity (mean = 0.768). The pairwise *t*-test between HAPPI-2’s sensitivity and STRING’s sensitivity returns *p*-value less than 10^−99^. The HAPPI-2’s sensitivity is also significantly higher than the BioGRID’s sensitivity (mean = 0.24). In the other hands, HAPPI-2’s rediscovery factor (*α*) (mean = 0.102) is also higher STRING’s *α* (mean = 0.099), with *p*-value = 3.56 × 10^−50^. Overall, we claim that although the superior of HAPPI-2 and STRING over BioGRID in rediscovery sensitivity could be due primarily to database extension with predictive PPIs, HAPPI could maintain better trade-off between discovery and expansion than STRING.

### Comparative evaluation of PPI data coverage vs quality tradeoffs

We evaluated the database’s potential for network biology applications, in comparison to all other constituent databases that were used to develop the HAPPI-2 database. In the Additional file 1: S1, we listed top 100 hub proteins from the HAPPI-2 along with each proteins’ node degree of connectivity and rank based on node degree of connectivity globally, for HAPPI-2', HAPPI-2, HAPPI-1', HAPPI-1, HPRD, I2D, IntenetDB, Wang’s dataset, BioGRID, and STRING. HAPPI-2’ and HAPPI-1’ refers to the medium- to ultrahigh-confidence subsets of the HAPPI-2 and HAPPI-1 database respectively. We observed that the ranking are not in consistent among all the databases, comprehensible given the varying data coverage and quality.

Using Spearman’s rank correlation, we also computed the all-vs-all pairwise correlation between the available database sources mentioned above (Table 3). From the table, we made several observations of the complex yet interconnected relationships of PPI hub data contents among the databases under evaluations. First, HAPPI-2 has the highest degree of rank correlation with other underlying constituent databases (using Spearman’s rank correlation coefficient ρ ≥0.25 as the threshold). Second, STRING’s network data content is similar to but different from that of the HAPPI-2 database (ρ =0.77), justifying our efforts in building the HAPPI-2 database. Third, BioGRID’s hub gene content is closer to HAPPI-2’ (a high-quality subset of HAPPI-2) with a ρ =0.65 than it does with STRING with a ρ =0.12. It is known that BioGRID collects PPIs primarily from validated experiments whereas STRING collects PPIs from both experimental and computational sources. Therefore, we claim that HAPPI-2, along with its unique PPI data quality ranking system, can help users balance the PPI data retrievals between high-coverage (STRING or HAPPI-2) and high biological support (BioGRID or HAPPI-2’) for themselves. Fourth, the HAPPI-2 and HAPPI-1 has decent correlation, although not quite high (ρ =0.40), suggesting that the connection for both data provenance and the content divergence between the two database releases. Fifth, the negative correlations, e.g., between IntNetDB and I2D (ρ = -0.60), and IntNetDB and BioGRID (ρ = -1.00), suggests that some of the database such as IntNetDB may capture dramatically different networks from the rest of the databases in comparison. One plausible explanation for the dramatic difference is the inherent high prediction errors from the IntNetDB and I2D databases.

**Table 3:** Calculated spearman’s rank correlation of PPIs among hub proteins found in common of each pair of human PPI databases under evaluation

	HAPPI-2’	HAPPI-2	HAPPI-1’	HAPPI-1	HPRD	I2d	IntNetDB	Wang	BioGRID	STRING
HAPPI-2’	**1.00**	**0.28**	0.19	−0.20	−0.07	0.57	0.23	0.05	**0.65**	0.19
HAPPI-2		**1.00**	**0.42**	**0.40**	0.06	**0.41**	**0.50**	0.01	0.02	**0.77**
HAPPI-1’			**1.00**	**0.51**	−0.12	−0.32	**0.60**	0.20	0.03	−0.11
HAPPI-1				**1.00**	−0.24	−0.60	**0.50**	−0.45	−0.21	−0.54
HPRD					**1.00**	**0.25**	0.00	**0.26**	−0.06	**0.55**
I2d						**1.00**	−0.60	−0.32	**0.56**	**0.49**
IntNetDB							**1.00**	0.00	−1.00	0.00
Wang								**1.00**	0.18	0.14
BioGRID									**1.00**	0.12
STRING										**1.00**

The Spearman’s correlation coefficient is calculated by comparing the rankings of network degrees of connectivity among top-100 highly connected HAPPI-2 human proteins in each pair of databases under comparison. HAPPI-2’ and HAPPI-1’ refers to the medium- to ultrahigh-confidence subsets of the HAPPI-2 and HAPPI-1 database respectively. The pairs of databases with high (>0.25) correlation coefficients are bolded

### Web-based database application

We have built a simple web-based application located at http://discovery.informatics.uab.edu/HAPPI with the goal of providing users with an intuitive web interface for easy access to human PPI data. In our design, we followed principles for bioinformatics application usability guidelines established by [74]. The web portal not only provide users with a simple interface to retrieve and filter results by key words or search criteria, but also enhance the retrieved results by providing detailed annotation of proteins and interactions including detailed functional descriptions of underlying proteins involved in the PPI, snapshots of protein family and disease annotation available. The advanced features are also provided to allow batch query and retrieval, filtering of data based on quality, and user content rating/annotations. In particular, we highlight the following features that have already been implemented (with a growing list to be updated periodically on the web site):

- Users can query the database with any gene symbol, UniProt ID, partial gene or protein name, or descriptions to search the database online.
- Users can search the database with a list of human gene or protein IDs to retrieve PPIs connected all within the list of genes or starting with the list of genes for one interaction neighborhood rings (in the Advanced Retrieval section).
- Users can explore information on the context of all drug targets or disease relevance among the PPI neighbors of the query genes/proteins.
- Users can personalize their interaction with the database content by optionally saving interactions in their accounts as user-managed PPI lists (Logon required only due to technical requirement of remembering user profiles).
- Users can now provide annotation comments or rate the quality of each PPI for future collaborative information filtering.
- Users can browse the proteins through the automatically annotated protein family and protein-disease categories to explore PPI data. All proteins are extensively hyperlinked with external public database reference.

**Fig. 8:**
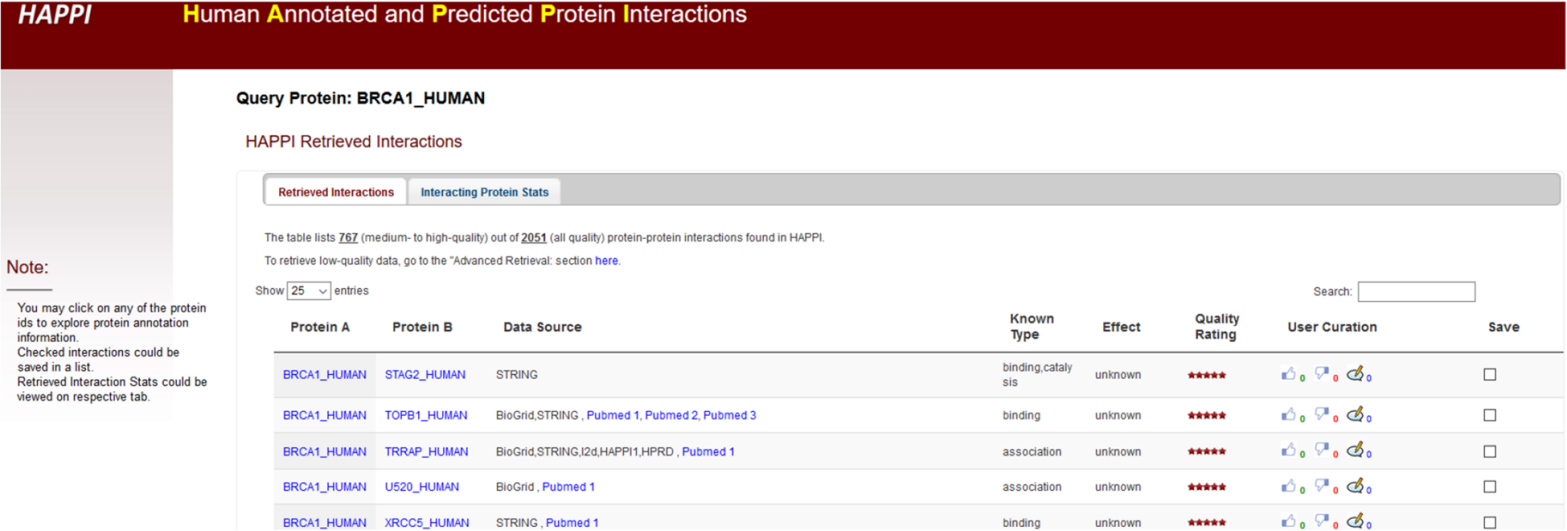
A Snapshot of the HAPPI 2.0 Web Interface Showing Partial Results of Protein Interactions Retrieved for the BRCA1 gene

An example of the retrieved interactions as a result of search for query protein BRCA1 gene. A snapshot of the human PPI user interface for the PPI page is shown in Fig. 8. The result table lists 767 (medium-to-high quality) out of 2,051 (all quality) protein-protein interactions found in the HAPPI-2 database and direct users to the advanced retrieval tab for low-quality data download for performance reasons. The users can type further key words into the search box at the upper right corner of the result table, or click on the table column headers to sort the fields in ascending or descending orders. Users may get additional protein functional details by clicking on the protein’s hyperlinked ID. Users may also select a subset of the PPI data retrieved to download using the “download” button provided, or to save the selections to the user account if necessary for future returned analysis. In addition, users can click on the “Interaction Stats” tab at the top of the result table to explore relevant drug-target protein and disease-gene relationships that may be available in the HAPPI-2 database.

## Discussion

Since the initial publication of HAPPI database in 2009, the surging interest in network biology and medicine has continued to fuel the growth of comprehensive public-accessible human PPI databases. Our goal is to develop a focused resource to enable users like ourselves—systems biologists—and other biology users to quickly retrieve information that is comprehensive in coverage, good in quality, well-annotated, and easy to use. We choose a comprehensive data integration approach to select some of the finest available databases such as BioGRID and STRING as the starting point for creating this valuable resource that addressed the data coverage and quality bias issues inherent in all the underlying databases. With the experimental feature to allow users to rate, annotate, and save contents as the database gains popularity in the future, HAPPI is poised to evolve itself into a useful resource for any users interested in human network biology and network medicine studies. We plan to keep updating the content periodically and implement additional features to make the database resource well integrated with the Bioconductor/R [56] or Cytoscape [75] downstream analysis in the future releases.

GO validation has been used as an approach to evaluate the quality of protein interaction data sets [76, 77], because experimentally validated PPIs tend to be stable interactions between proteins performing similar GO functions. However, due to the potential bias of data sets that predict PPIs based on GO similarity, it is possible that subsets of the PPI database being validated are enriched with PPIs with high GO similarity. This is unlikely to be the case for the new HAPPI database, because our Fig. 7 shows that the predicted PPIs would have a low reliability index approximately equivalent to low-quality PPIs unless confirmed with additional data sources. On the other hand, the BioGRID database, which was primarily curated from literature or trusted experimentations, has a relatively high GO similarity profile.

In this paper, we setup the starting system in HAPPI-1 and HAPPI-2 based on the arbitrary choice of parameters; therefore, it lacks of universal justification. The usefulness of our HAPPI-1 and HAPPI-2 reliability index is problem/question-specific. In this paper, we use our reliability index in some specific tasks, such as GO annotation and true-positive validation with MetaGene. The reportable outcomes in this paper justify the reliability index, but only within the practices inside this paper. However, we do not guarantee that the reliability index could be useful in other problems. We believe that the users are responsible to decide how to use and modify our reliability index in specific researches.

## Availability and requirements 

**Project name:** HAPPI version 2.0

**Project home page:** http://bio.informatics.uab.edu/HAPPI/

**Operating system:** Any version of Windows/MacOS/Linux/Unix, with a standard web browser

**Other requirements:** None

**License:** free for non-commercial use

## Additional file

Additional file 1: Gene Ontology (GO) term analysis in HAPPI-2. (PDF 113 kb)

## Abbreviations

GO: Gene ontology
HAPPI: Human annotated and predicted protein interactions
PPI: Protein-protein interaction
