# Supplementary material for: HAPPI-2: a Comprehensive and High-quality Map of Human Annotated and Predicted Protein Interactions
Source: BMC Genomics. 2017 Feb 17;18:182. doi: 10.1186/s12864-017-3512-1 (PMC5314692; doi:10.1186/s12864-017-3512-1)
Supplement: Additional file 1: — Gene Ontology (GO) term analysis in HAPPI-2. (PDF 113 kb) [file 12864_2017_3512_MOESM1_ESM.pdf]

ProteinName	HAPPI2 DEGREE	HAPPI2 RANK	HAPPI2-3STAR DEGREE	HAPPI2-3STAR RANK	HAPPI1 DEGREE	HAPPI1 RANK	HAPPI1-3STAR DEGREE	HAPPI1-3STAR RANK	STRING DEGREE	STRING RANK	I2D DEGREE	I2D RANK	INTNETDB DEGREE	INTNETDB RANK	WANG DEGREE	WANG RANK	HPRD9 DEGREE	HPRD9 RANK
UBC_HUMAN	9532	1	9355	1	0	NA	0	NA	14006	3	1246	1	0	NA	121	275	23	1119
TSPOA_HUMAN	4631	2	698	39	0	NA	0	NA	9340	4	2	11542	0	NA	0	NA	0	NA
ALBU_HUMAN	4416	3	820	25	330	1584	44	3082	8722	5	173	407	121	2775	3	4404	19	1422
INSR2_HUMAN	4214	4	849	21	0	NA	0	NA	8492	6	0	NA	0	NA	0	NA	0	NA
AKT1_HUMAN	3874	5	1046	13	1256	89	354	53	7182	8	218	270	250	1072	1410	5	192	22
RL40_HUMAN	3854	6	845	23	34	7025	24	4832	7602	7	319	107	0	NA	115	310	26	952
DPOLA_HUMAN	3488	8	631	66	522	697	216	268	6588	10	131	626	339	625	15	2278	23	1119
HCDH_HUMAN	3488	8	395	270	5220	7	456	17	2250	284	9	6675	110	3071	1	6075	4	5581
G3P_HUMAN	3487	9	559	86	874	186	82	1518	6542	11	263	177	143	2316	21	1888	56	274
CDK2_HUMAN	3442	10	1068	12	3872	19	562	4	3240	82	217	274	211	1387	366	52	114	72
P53_HUMAN	3433	11	1206	8	1412	69	572	3	6122	15	827	5	335	640	1000	9	412	1
A4_HUMAN	3400	12	2388	2	438	981	136	702	3122	96	261	184	251	1061	45	1045	119	66
RASH_HUMAN	3330	13	546	98	1146	108	338	61	6238	13	133	613	102	3302	204	115	95	107
B4GT4_HUMAN	3325	14	286	658	6492	1	552	5	174	12255	0	NA	8	8545	0	NA	0	NA
DPOD1_HUMAN	3285	15	525	111	156	3834	118	892	6450	12	79	1132	426	403	21	1888	10	2837
JUN_HUMAN	3281	16	814	26	1960	45	470	12	5624	18	262	182	144	2300	359	54	176	30
NSDHL_HUMAN	3279	17	295	610	6216	2	512	9	392	7155	42	2211	70	4531	0	NA	0	NA
PKN1_HUMAN	3278	18	433	204	4462	11	428	24	2170	316	37	2499	867	23	36	1250	50	339
ECHA_HUMAN	3169	19	347	386	5586	5	516	8	672	4041	44	2105	423	408	0	NA	0	NA
PPB1_HUMAN	3132	20	396	266	1162	103	256	148	5382	19	7	7619	37	6240	27	1559	4	5581
TNFA_HUMAN	3122	21	795	29	450	940	86	1433	5908	17	87	1028	256	1031	1600	2	19	1422
DPOLZ_HUMAN	3102	22	340	401	94	5206	28	4378	6170	14	3	10427	202	1475	0	NA	5	4859
DPOE1_HUMAN	3093	23	454	178	322	1655	148	610	5958	16	121	690	370	516	17	2150	11	2588
ADH1A_HUMAN	3036	24	280	692	5494	6	464	13	716	3739	6	8149	111	3040	2	5019	2	7675
B4GT2_HUMAN	3006	25	254	987	5736	4	444	19	240	10227	1	13156	24	7160	0	NA	0	NA
ADH1G_HUMAN	2980	26	213	1453	5838	3	422	27	0	NA	4	9587	134	2509	0	NA	2	7675
ODB2_HUMAN	2950	27	278	705	5034	8	522	7	1144	1737	11	6008	14	7977	11	2600	0	NA
SRC_HUMAN	2914	28	916	17	1160	104	364	48	5302	22	529	25	57	5120	450	38	309	4
CALM_HUMAN	2902	29	1420	3	572	562	204	316	14718	2	551	22	178	1788	401	43	184	26
HXK4_HUMAN	2838	30	308	537	4852	9	442	20	1074	1981	9	6675	28	6880	42	1109	5	4859
CDK9_HUMAN	2777	31	576	81	4316	14	454	18	1138	1760	189	364	613	119	58	813	50	339
FINC_HUMAN	2771	32	1088	11	360	1405	30	4184	4314	41	522	28	177	1796	156	176	90	121
CD4_HUMAN	2738	33	583	78	494	782	198	342	5304	21	47	1978	30	6739	212	108	36	574
ESR1_HUMAN	2726	35	909	18	908	168	218	258	5052	27	588	15	59	5018	184	136	281	7
MPIP1_HUMAN	2726	35	461	168	4178	16	528	6	1794	576	72	1268	106	3184	41	1135	41	462
EGFR_HUMAN	2699	37	948	15	736	297	300	90	4760	36	548	23	164	1973	397	44	236	13
MYC_HUMAN	2699	37	1282	6	1514	61	292	102	4538	38	893	4	293	824	984	11	121	63
TGFB1_HUMAN	2697	38	667	49	670	381	136	702	4832	33	56	1625	403	438	393	45	44	405
PPBT_HUMAN	2694	39	313	513	210	2876	58	2359	5310	20	28	3171	12	8138	0	NA	4	5581
KPCE_HUMAN	2690	40	476	152	4296	15	424	26	1162	1681	225	258	39	6112	156	176	59	250
NRF1_HUMAN	2685	41	55	6239	218	2735	52	2612	460	6223	4	9587	2	9420	1	6075	8	3510
PCNA_HUMAN	2684	42	744	36	564	584	256	148	5084	26	296	133	201	1486	64	708	128	57
CASP3_HUMAN	2683	43	510	121	1636	54	318	75	4268	45	188	365	212	1380	198	119	190	24
TYRO_HUMAN	2669	44	287	651	314	1712	44	3082	5250	23	4	9587	28	6880	8	3051	1	9186
FOS_HUMAN	2625	45	686	43	688	358	174	460	4824	34	162	458	179	1772	456	35	92	115
PPBN_HUMAN	2609	46	303	559	70	5836	58	2359	5172	25	21	3941	58	5082	0	NA	3	6475
CKAP2_HUMAN	2595	47	256	885	0	NA	0	NA	5230	24	2	11542	0	NA	0	NA	1	9186
PTEN_HUMAN	2547	48	450	182	730	307	164	522	4832	33	86	1038	132	2545	86	476	42	447
AL3A2_HUMAN	2543	49	276	718	4720	10	434	22	404	6988	7	7619	91	3661	0	NA	0	NA
IL6_HUMAN	2527	51	579	80	730	307	256	148	4840	31	18	4386	43	5885	214	106	6	4305
VEGFA_HUMAN	2527	51	580	79	474	845	100	1162	4898	29	46	2019	148	2219	428	40	28	860
RAN_HUMAN	2514	52	637	61	540	655	134	713	4014	52	239	222	1219	1	128	246	66	201
KPCD3_HUMAN	2491	53	232	1271	4336	13	360	49	688	3915	15	4971	200	1502	1	6075	8	3510
EGF_HUMAN	2490	54	426	217	516	720	56	2441	4764	35	24	3556	56	5201	192	124	21	1269
CHK1_HUMAN	2481	55	631	66	2736	32	408	29	2586	184	117	720	320	694	59	805	36	574
ELAV1_HUMAN	2468	56	245	1171	452	925	66	2014	4390	39	38	2447	283	858	10	2734	18	1523
MK01_HUMAN	2451	58	921	16	1100	118	320	70	4036	51	392	62	336	637	1170	7	244	11
NOTC1_HUMAN	2451	58	477	147	882	180	174	460	4292	44	77	1166	125	2683	390	46	71	182
KPCD_HUMAN	2411	59	526	109	3690	22	426	25	1114	1847	142	552	355	565	191	125	153	39
HSP74_HUMAN	2381	60	510	121	724	315	136	702	4266	46	43	2155	265	966	34	1298	26	952
DYH8_HUMAN	2351	61	239	1210	0	NA	0	NA	4752	37	0	NA	0	NA	7	3220	0	NA
HS90A_HUMAN	2328	62	1272	7	846	208	276	118	2716	158	968	3	184	1706	507	31	130	55
DGKI_HUMAN	2327	63	228	1311	4426	12	418	28	310	8557	3	10427	2	9420	0	NA	0	NA
ERBB2_HUMAN	2302	64	416	233	792	239	248	166	4310	42	263	177	82	4020	125	256	72	175
CYC_HUMAN	2293	66	365	334	336	1549	80	1583	4324	40	62	1472	234	1192	37	1223	22	1196
IFNG_HUMAN	2293	66	418	229	768	263	164	522	4300	43	7	7619	90	3710	319	66	8	3510
KC1G3_HUMAN	2272	67	261	810	3820	20	398	37	740	3580	1	13156	241	1130	20	1961	1	9186
CCND1_HUMAN	2239	68	522	113	1058	126	236	199	4172	49	129	636	12	8138	376	51	57	271
KPYM_HUMAN	2228	69	562	84	2102	43	458	16	2488	197	204	311	108	3126	16	2213	0	NA
CHK2_HUMAN	2213	70	369	328	2726	33	400	35	1994	423	67	1373	100	3367	55	866	58	265
STAT3_HUMAN	2210	71	621	68	1316	81	406	30	3550	60	240	220	305	754	538	27	155	38
MP2K4_HUMAN	2158	72	338	414	3210	26	484	11	1422	1094	42	2211	72	4441	110	339	32	701
CSKP_HUMAN	2136	73	464	166	2774	30	430	23	1542	891	136	592	281	872	6	3453	72	175
PLCD1_HUMAN	2112	74	298	589	3960	17	502	10	360	7652	9	6675	67	4646	12	2512	10	2837
MCM8_HUMAN	2107	75	244	1177	32	7103	10	6641	4206	48	5	8771	0	NA	0	NA	3	6475
ABCE1_HUMAN	2105	77	393	275	312	1720	54	2527	3758	57	6	8149	590	147	0	NA	3	6475
FRIH_HUMAN	2105	77	322	471	154	3876	56	2441	4050	50	96	916	117	2884	7	3220	13	2208
H33_HUMAN	2087	78	718	37	244	2386	36	3661	7144	9	519	29	11	8166	43	1083	20	1342
GAK_HUMAN	2071	79	325	462	3256	25	458	16	690	3900	18	4386	542	190	2	5019	17	1645
GRB2_HUMAN	2062	80	1145	10	1142	109	378	44	3300	75	1156	2	301	778	738	16	288	6
B4GT6_HUMAN	2050	81	204	1549	3880	18	386	41	232	10448	0	NA	6	8799	0	NA	0	NA
CSN5_HUMAN	2040	82	983	14	40	6800	40	3351	2700	162	234	235	803	30	41	1135	54	291
RAC1_HUMAN	2036	83	658	55	1090	120	288	106	3284	77	247	204	428	396	1732	1	135	51
PK3CA_HUMAN	2035	84	675	44	560	590	240	187	3526	61	53	1724	230	1229	455	36	28	860
REL_HUMAN	2022	85	265	776	680	367	130	758	3356	70	74	1230	554	171	16	2213	33	669
SODM_HUMAN	2021	86	304	551	474	845	84	1475	3766	56	97	902	90	3710	23	1789	8	3510
MD2L2_HUMAN	1998	87	235	1246	3110	27	374	47	1010	2197	33	2762	0	NA	21	1888	20	1342
BRCA1_HUMAN	1990	88	747	34	764	269	232	211	3346	71	256	193	263	983	85	481	178	29
MK03_HUMAN	1978	89	619	69	1392	72	382	43	3020	109	191	354	15	7829	1084	8	165	37
CDN1A_HUMAN	1974	90	549	95	990	144	196	357	3388	67	294	137	200	1502	516	29	75	159
TESK1_HUMAN	1973	91	221	1376	3798	21	398	37	138	13460	12	5693	42	5964	6	3453	12	2389
SODC_HUMAN	1956	92	240	1203	618	468	66	2014	3468	63	137	584	104	3241	16	2213	12	2389
STK39_HUMAN	1943	93	207	1515	3570	23	346	58	334	8108	18	4386	50	5533	9	2889	14	2046
PRP4B_HUMAN	1942	94	264	788	1674	52	278	116	2090	354	24	3556	459	328	12	2512	7	3873
ACTG_HUMAN	1938	95	472	157	532	672	88	1385	3218	83	451	41	381	491	78	568	46	376
IGF1_HUMAN	1918	96	350	374	0	NA	0	NA	3856	54	19	4216	0	NA	200	116	17	1645
NGF_HUMAN	1912	97	311	520	276	2017	34	3827	3746	58	19	4216	6	8799	110	339	14	2046
CADH1_HUMAN	1907	99	411	242	284	1939	98	1192	3694	59	66	1384	75	4280	230	95	58	265
CASA1_HUMAN	1907	99	190	1718	0	NA	0	NA	3786	55	6	8149	74	4347	3	4404	4	5581
HDAC1_HUMAN	1905	100	760	32	728	311	300	90	3072	103	415	54	514	241	126	252	190	24
